# Thoughts on Dental Caries

**Published:** 1860-11

**Authors:** Geo. Watt

**Affiliations:** Written for the Mad River Valley Society, October


### THOUGHTS ON DENTAL CARIES.

BY GEO. WATT.

Written for the Mad River Valley Society, October.

DENTAL caries, as ordinarily used, is a misnomer; yet it is more convenient to retain, than to abandon its use. A man who has all his life called himself Smith, will find it inconvenient to do otherwise, even when he has discovered that his real name is Thompson. The term dental caries was in general use, to designate several distinct morbid conditions of the teeth, before we had any accurate knowledge of their nature. And as the principal phenomena of these conditions are well known, and are easily observed, there is little likelihood of mistakes occurring in regard to the application of the term. The term caries is usually applied to mortification, or rather, to ulceration of bone; and it was applied to decay of the teeth, under the supposition that it results from inflammatory action. For want of a better term, we still continue it, finding it easier to modify the definition than to change the word.

It is now admitted, by all who are familiar with the subject, that, whatever may be the predisposing causes, the immediate cause of dental caries is chemical action. It is well known that constitutional causes have much to do with this disease, both in producing badly organized, defective teeth, and in eliminating, or preparing the agents which act chemically on them. But no constitution produces teeth so defective that they undergo spontaneous decomposition, while retaining a vital connection with the general system. I am aware that a few pathologists still maintain that inflammation of the bony texture of the teeth, is liable to the same terminations as inflammation of ordinary bony tissue; but it is not profitable to debate this point in the present paper. Suffice it to say that the structure and position of the enamel indicate that the danger is from without, not from within.

As soon as it is admitted that decay of the teeth results from chemical action, it is natural to inquire what agent, or agents produce this action. Accordingly, we find the profession turned at once in this direction. And when the composition of the teeth is taken into the account, we would infer that the deleterious agents are to be looked for among the acids. And here we have had great confusion of ideas, and are likely still to have it. For example, we are told "that it is proven that nearly all the acids, both mineral and vegetable, act readily upon the teeth." (Harris' Dictionary, Art. "Caries of the teeth.") Upon any part of the teeth? Or, are we to understand, that some of them act on the animal portion, some on the earthy, and some, or all, on the enamel? Just turn to the index of almost any chemical text book, and ask yourself it if is proven that nearly all of the acids there named act readily upon the teeth. Do carbonic acid, tannic acid, and scores of others that might be named, act *readily* upon the teeth? This expression, and many others, that might be quoted from various writers, show a professional longing for, rather than an attainment of the truth in regard to this matter.

Now, for convenience, let us assume that dental caries is produced by the action of acids. The question still arises, what acids? Are many acids, or only a few, concerned in its production? One of the laws of combination teaches us that chemical compounds are definite in their nature. Chemical action is always definite. When an acid combines with an alkali, or base, a definite compound, called a salt, is formed. When a different acid unites with this same base, a different salt is formed. Each salt, each chemical compound of any kind, is distinguished from all others, by characteristics peculiar to itself. It is unlike all other substances, in some respects. Each chemical result differs from all other chemical results. Of course, then, a great variety of chemical reagents will produce a great variety of chemical reactions.

Let us now inquire as to the various characteristics of those chemical actions which result in what we recognize as dental caries. Do we here find a great variety of appearances? Or is it not well known that the phenomena of caries are so few, and so circumscribed, that, by common professional consent, but three or four varieties of it are recognized? We find one variety often called "white decay," and another that is brownish in color, and a third, that is very properly designated as "black decay." These differ in other respects, as well as in color. In the white variety all the components of the teeth are acted on, and disintegrated, as far as the disease extends. In the second variety, the earthy portion of the tooth seems to be removed, while much, or all of the animal portion remains, which is conclusive evidence that the chemical agent, whatever it may be, forms soluble compounds with the earthy materials. In the "black decay" there is less disintegration of the tooth substance than in either of the other varieties; and it progresses less rapidly than either of them. The physical characteristics of this variety, aside from the chemical, would indicate that the chemical agent, principally concerned in its production, forms, mainly, insoluble compounds with the constituents of the tooth. Then, there is a fourth variety, commonly called "chemical abrasion," in which the entire tooth substance is *removed*, as far as the disease extends. It is evident that the agent producing this, dissolves, or forms soluble compounds with both the animal and earthy materials of the tooth.

Unless we conclude that chemical compounds are not definite in their nature, and that many reagents may produce but a few reactions, we are forced to the conclusion that dental caries, as observed and recognized, results from the action of but few substances on the teeth. It is very probable that each *distinct* variety is produced by the action of a single agent, and, invariably, by the same agent. I am well aware that more than one variety may be found in the same mouth, at the same time, and in close proximity; and consequently, any given case of caries may partake of the characteristics of more than one variety. It is not uncommon to find "white decay" attacking a tooth in a cavity primarily affected with the brown, or colorless variety. But every practitioner is familiar with unmixed cases, representing all the four classes specified.

The physical characteristics of decay depend much on the the texture of the teeth affected; but they are dependent, also, on the nature of the compounds formed by the union of the destroying agent with the constituents of the teeth. The degree of concentration of the chemical agent has also a modifying influence. When much diluted its action is almost solely in obedience to its strongest affinity. For example, if nitric acid were the agent, when concentrated, it would act energetically on the animal, as well as on the earthy materials of the teeth; but when much dilated its action would be almost confined to the latter.

The chemical characteristics of decay, however, depend almost exclusively on the character of the agent producing it. The truth of this appears evident, when we reflect that bad teeth and good ones are composed of the same chemical substances. Marble and chalk are alike in chemical composition, but not in physical structure; and, though an acid, acts more rapidly on the latter than on the former, yet the result of the action is the same. An acid, too, will act with more energy on a soft, porous tooth, than on one of firmer texture; yet the chemical results are the same. It is safe to conclude, then, that as there are but few *results* in the chemical actions attendant on dental caries, there are but few chemical agents immediately concerned in their production.

It is not to be inferred from the above that but few agents are capable of injuring the teeth by chemical action. Many acids used in food, or as medicines, are capable of doing injury to the teeth. But no one need suppose that an acid, even though considerably concentrated, brought occasionally in contact with the teeth, is the immediate cause of caries.

Every close observer will conclude that caries is the result of an agent acting slowly and steadily in the accomplishment of its work. He will be apt to infer that this agent is either formed by chemical action within the mouth, or is eliminated therein, either as a secretion or an excretion, and that it quietly performs its disastrous deeds as fast as formed or eliminated. The application to the teeth, of an acid capable of acting chemically on them, facilitates, or predisposes to, the production of caries; and this it may do, without this acid being the *immediate* cause of the decay. A tooth may be fractured, or its enamel may be removed, by mechanical means; and, as the dentine is thus exposed, the tooth is more liable to caries than before the exposure. But no one supposes that the mechanical action which exposes the dentine is the immediate cause of the caries. The dentine would remain sound and healthy, did not some chemical agent attack it. In like manner, in the administration of acids, as food or medicine, the teeth may be so corroded as to expose the dentine, and render it as liable to the action of the carious agent as in the former case; or it the dentine is not exposed, the enamel may be roughened, either mechanically or chemically so as to afford a lodgment for organic matter, which by decomposition, may generate one of the acids immediately concerned in the production of caries. On this principle, acid medicines and acid foods may indirectly, but not immediately, cause caries. The same remarks will apply to acids brought in contact with the teeth by eructation or vomiting.

If this view is correct, the investigation of the subject of dental caries is brought within a narrower compass than many suppose. The first step is to inquire what acids, in health and disease, are liable to be secreted or excreted, so as to be brought regularly in contact with the teeth. The second is to ascertain what acids are liable to be formed within the mouth, by fermentation, or otherwise. And the third is to discover what ones of all these, are capable of producing the phenomena of dental caries. There is but little room to doubt that, at least, each of the first three varieties is the result of a *specific* agent. And if these unstudied remarks should lead others to investigate this matter we will be satisfied.

